# Prevalence of the MDR1 gene mutation in herding dog breeds and Thai Ridgebacks in Thailand

**DOI:** 10.14202/vetworld.2021.3015-3020

**Published:** 2021-11-27

**Authors:** Chommanad Lerdkrai, Nuch Phungphosop

**Affiliations:** Department of Physiology, Faculty of Veterinary Medicine, Kasetsart University, Bangkok, Thailand

**Keywords:** herding dog breeds, multi-drug resistance 1 gene, mutation, P-glycoprotein, Thai Ridgeback dogs

## Abstract

**Background and Aim:**

A canine multi-drug resistance 1 (MDR1) nt230(del4) is a well-known inherited disorder that primarily affects collies and various herding breeds. The most recognized clinical implication for affected dogs is associated with an increased risk of multiple drug toxicity. To date, MDR1 gene mutations have been identified globally, especially in dogs from the USA and European countries. This study aimed to investigate the prevalence of MDR1 nt230(del4) in herding dog breeds and Thai Ridgebacks in Thailand.

**Materials and Methods:**

We clarified the prevalence of MDR1 nt230(del4) in 263 dogs of eight purebred dog breeds in Thailand using an allele-specific multiplex polymerase chain reaction method and direct DNA sequencing.

**Results:**

Rough Collies, Australian Shepherds, Shetland Sheepdogs, and Old English Sheepdogs were affected by the mutation with mutant allelic frequencies of 57.14%, 12.82%, 11.28%, and 8.33%, respectively. Among these populations, the prevalence of the MDR1 (+/–) genotype was 57.14% (12/21) for Rough Collies, 25.64% (10/39) for Australian Shepherds, 16.13% (15/93) for Shetland Sheepdogs, and 16.67% (2/12) for Old English Sheepdogs, whereas the MDR1 (–/–) mutation was only identified in Rough Collies and Shetland Sheepdogs, with prevalences of 28.57% (6/21) and 3.22% (3/93), respectively. However, the MDR1 nt230(del4) was not identified in Border Collies, German Shepherds, White Swiss Shepherds, or Thai Ridgebacks.

**Conclusion:**

This study provides the current situation regarding MDR1 nt230(del4) in herding dog breeds in Thailand. In this survey, we investigated for the first time the status of MDR1 genotype in Thai Ridgebacks. These results are helpful for veterinarians managing effective therapeutic plans for commonly affected dog breeds, and these results will encourage all breeders to improve their selective breeding programs based on the MDR1 nt230(del4) status.

## Introduction

The multi-drug resistance 1 (MDR1) gene, the so-called ABCB1 gene, encodes a transmembrane transporter P-glycoprotein, which is a constituent of the membrane-bound ATP-binding cassette superfamily. P-glycoprotein is widely distributed across different tissues and physiological barriers, such as the blood-brain and blood-testis barriers [1-3]. Pharmacokinetic studies have shown that P-glycoprotein controls the metabolism of its substrates and promotes the elimination of xenobiotics [4,5]. In 1980, the ivermectin-sensitive phenotype was first reported in collies, and life-threatening intoxication occurred because of the accumulation of ivermectin in the central nervous system [6]. A later study in 2001 showed that the underlying cause of this toxicity is associated with a mutation in the MDR1 gene. The MDR1-deficient genotype involves a 4-base pair deletion at nucleotide position 230 in the fourth exon of the MDR1 gene (MDR1 nt230[del4]), which generates a shift in the reading frame and leads to a premature stop codon, and this results in a severely shortened and nonfunctional P-glycoprotein, which disturbs the integrity of the blood-brain barrier [6,7].

In addition to collies breeds, the MDR1 nt230(del4) has been described in other breeds, including Shetland Sheepdogs, Border Collies, Australian Shepherds, Miniature Shepherds, Old English Sheepdogs, English Shepherds, German Shepherds, White Swiss Shepherds, McNabs, Wällers, and Longhaired Whippets [8,9]. Dogs harboring the mutated MDR1 genotype are sensitive to the toxic effects of certain P-glycoprotein substrates, including ivermectin, moxidectin, doramectin, loperamide, digoxin, acepromazine, ondansetron, vincristine, vinblastine, doxorubicin [8-10], and non-specific P-glycoprotein substrates, such as apomorphine [11]. The toxicity risk depends on the number of contributing mutant alleles. Homozygous dogs that carry two copies of the mutated MDR1 gene tend to develop more severe clinical signs due to drug intoxication than heterozygous dogs that possess a single copy of this mutation [6,12].

The distribution of MDR1 nt230(del4) has been identified in many countries for decades. In 2012, the preliminary study of MDR1 genotyping was first reported in Thailand by Asawakarn *et al*. [13]. In the 43 dogs of four different herding breeds evaluated, the presence of MDR1 nt230(del4) was observed in Rough Collies and Shetland Sheepdogs, while Border Collies and German Shepherds were free of the mutation. During the past decade, there has been a notable increase in the number of herding dog breeds in Thailand due to a growing tendency of importing dogs from abroad. As screening of the MDR1 genotype is not routinely performed in our country, the current mutation status of the MDR1 gene in Thailand remains largely unexplored, especially in the most prone herding and native breeds.

This study aimed to investigate the prevalence of MDR1 nt230(del4) in herding breeds potentially affected, including Rough Collies, Border Collies, Shetland Sheepdogs, Old English Sheepdogs, Australian Shepherds, German Shepherds, and White Swiss Shepherds. In addition, a native Thai Ridgeback was included in the survey.

## Materials and Methods

### Ethical approval

The research was conducted under Animal Use Protocol number ACKU60-VET-017, approved by the Kasetsart University Institutional Animal Care and Use Committee.

### Study period and location

This research was conducted from February 2018 and December 2020. The study was conducted at the Department of Physiology, Faculty of Veterinary Medicine, Kasetsart University, Thailand.

### Animals and sample collection

Venous blood samples were collected on a voluntary basis from 263 clinically healthy dogs from the central part of Thailand. In total, all samples with unknown MDR1 status were obtained under consent from seven breeding kennels (one kennel for Border Collies, two for Shetland Sheepdogs, two for Australian Shepherds, and two for Thai Ridgebacks) and 96 private dog owners that presented at Kasetsart University Veterinary Teaching Hospital. This survey evaluated data from eight purebred dog breeds: 21 Rough Collies, 93 Shetland Sheepdogs, 45 Border Collies, 39 Australian Shepherds, 20 German Shepherds, 12 Old English Sheepdogs, four White Swiss Shepherds, and 29 Thai Ridgebacks, with or without pedigree data or breeding information. The characteristics for each breed were identified according to the pedigree and/or the visual appearance.

### Polymerase chain reaction (PCR)-based diagnostic of the MDR1 genotype

Genomic DNA was extracted from ethylenediaminetetraacetic acid whole blood using the QIAmp DNA Blood Mini Kit (Qiagen GmbH, Germany) following the instructions provided and preserved at −20°C until further analysis. An allele-specific multiplex PCR-based assay was performed for detecting the wild-type allele and the mutant MDR1 allele as previously described with minor modifications [14]. Briefly, the wild-type allele and the mutant MDR1 allele were tested in each sample with two separate PCR reactions using different specific primer sets (Table-1). The PCR 1 primer set, consisting of the forward primer PgpA, the forward wild-type allele-specific primer PgpB, and the reverse primer PgpD, was used to identify the wild-type MDR1 allele. The PCR 2 primer set, consisting of the forward primer PgpA, the forward mutant allele-specific primer PgpC, and the reverse primer PgpD, was used to identify the mutant MDR1 allele. This method provided the co-amplification of a 483 or 479 bp internal control amplicon and a 326 bp allele-specific amplicon. All PCR reactions were conducted in a final reaction volume of 25 μL consisting of 1× High Fidelity reaction buffer (10×, Invitrogen, USA), 0.2 mM each dNTP (Biotechrabbit, Germany), 2.0 mM MgSO_4_ (Invitrogen), 0.2 μM for each primer (Biobasic Inc., Canada), and 1 U Platinum. *Taq* DNA Polymerase High Fidelity (Invitrogen) and 50-70 ng of genomic DNA template. The amplification was performed in a T100 Thermal Cycler (Bio-Rad, USA) with the following protocol: Initial denaturation for 5 min at 95°C, 35 amplification cycles (denaturation for 30 s at 95°C, annealing for 2 min at 57°C for the detection of the wild-type allele or at 61°C for detection of the mutant allele, and an extension step for 1 min at 72°C), and a final extension step for 10 min at 72°C. The amplified products were analyzed on 1.5% agarose gel electrophoresis, staining with ethidium bromide, and visualized under ultraviolet transillumination.

**Table-1:** Details of the primer sequences used for allele-specific multiplex PCR.

Primer name	Nucleotide sequence 5’ to 3’
PgpA	Forward primer: CATGAAACTGTGCTAATTTCC
PgpB	Forward primer: TTGGAAACATGACAGATAGC
PgpC	Forward primer: GTTTTTGGAAACATGACAGC
PgpD	Reverse primer: AACTTCCTGGGATCTTTCTG

PCR=Polymerase chain reaction

### Sequencing

A total of 263 samples were submitted for direct DNA sequencing. A fragment containing the target site of MDR1 nt230(del4) was amplified using primers PgpA and PgpD. The PCR amplification protocol was identical to that used for the multiplex PCR, other than using an annealing temperature of 58°C [14]. The PCR products were purified with a NucleoSpin^®^ Gel and PCR Clean-up purification kit (Macherey-Nagel, Germany) according to the manufacturer’s instructions and then sequenced by Macrogen Inc. (Korea) using the reverse primer. Data were analyzed using Unipro UGENE software. The sequence similarity was compared with the database reported in the National Center for Biotechnology Information (NCBI; https://blast.ncbi.nlm.nih.gov/Blast.cgi).

## Results

The results of MDR1 genotyping and mutation frequency are summarized in Table-2. From the eight dog breeds surveyed, a total of 263 samples were obtained from five herding breeds related to the collie lineage (Rough Collies, Shetland Sheepdogs, Australian Shepherds, Old English Sheepdogs, and Border Collies), two herding breeds not closely related to the collie (White Swiss Shepherds and German Shepherds), and Thai Ridgebacks. The MDR1 nt230(del4) was only noted in Rough Collies and the collie-related breeds, other than Border Collies. Conversely, the MDR1 nt230(del4) was not detected in German Shepherds, White Swiss Shepherds, or Thai Ridgebacks. Among the four breeds carrying MDR1 nt230(del4) in this survey, the mutation frequency was highest in Rough Collies (57.14%), followed by Australian Shepherds (12.82%), Shetland Sheepdogs (11.28%), and Old English Sheepdogs (8.33%). In all Rough Collies and Shetland Sheepdogs examined, the occurrence of either the MDR1 (+/–) or the MDR1 (–/–) genotype was identified. When comparing the pattern of MDR1 nt230(del4) in mutated cases, the MDR1 (+/–) genotype occurred more frequently than the MDR1 (–/–) genotype. In Australian Shepherds and Old English Sheepdogs studied, all mutated cases exhibited the MDR1 (+/–) genotype pattern, and the MDR1 (–/–) genotype was not observed in either of these breeds.

**Table-2:** MDR1 genotyping and mutant allelic frequency in 263 dogs of eight breeds in Thailand.

Breed (N)	MDR1(+/+)		MDR1(+/–)		MDR1(–/–)		Mutant allelic Frequency (%)
		
	n	% genotype	n	% genotype	n	% genotype	
Rough Collie (21)	3	14.29	12	57.14	6	28.57	57.14
Australian Shepherd (39)	29	74.36	10	25.64	0	0	12.82
Shetland Sheepdog (93)	75	80.65	15	16.13	3	3.22	11.28
Old English Sheepdog (12)	10	83.33	2	16.67	0	0	8.33
Border Collie (45)	45	100	0	0	0	0	0
German Shepherd (20)	20	100	0	0	0	0	0
White Swiss Shepherd (4)	4	100	0	0	0	0	0
Thai Ridgeback Dog (29)	29	100	0	0	0	0	0

MDR1 (+/+)=Homozygous wild-type genotype, MDR1 (+/–)=Heterozygous mutation, MDR1 (–/–)=Homozygous mutation genotype, N=Number of tested dogs of each, n=Number of dogs of each genotypic pattern.

## Discussion

In this research, we characterized the current prevalence of MDR1 nt230(del4) in herding dog breeds and Thai Ridgebacks in Thailand. In a previous study in 2012 [13], the highest mutation frequency of 71.9% was reported in Rough Collies, and this frequency was remarkably higher than that of other related sheepdogs. Interestingly, a similar trend was identified in our survey, although with a slightly lower mutation frequency. These findings indicate that the prevalence of MDR1 nt230(del4) has remained relatively high in the Rough Collie population over several years. As mentioned by the previous study, a low mutation frequency of 5.5% was found in a small group of Shetland Sheepdogs. However, within a larger population of the same breed investigated in the current study, this mutation frequency was about 2-fold higher. The progressive increase in the rate of MDR1 nt230(del4), compared to the previous study in 2012, is presumably due to the notable increase in the Shetland Sheepdog population as this breed becomes more popular in our country. In the previous research, all evaluated Border Collies and German Shepherds exhibited 100% of the intact MDR1 (+/+) genotype, and this result is comparable to the findings of the present survey. Therefore, we speculate that the prevalence of MDR1 nt230(del4) is relatively low in Border Collies and German Shepherds and has remained constant over the past decade in Thailand.

In comparing our results to MDR1 nt230(del4) trends from global research, we have identified several interesting trends. Of Rough Collies evaluated in this survey, the privately-owned dogs originated mainly from the USA, with some originating from Russia and Uzbekistan. Almost all individuals descended from different parental lineages, other than four dogs that shared the same parental pair. Although the population of Rough Collies in the current survey was relatively small, as it is an unusual dog breed in Thailand, the current results are similar to those from large-scale studies in the USA, where the mutation frequency was 54.6-56% [15,16]. Moreover, our findings agree with those from several studies from Japan [17], Brazil [18], South Africa [19], Australia [20], Germany [14,21,22], France [23], the UK [24], Belgium [25], Italy [26], and numerous European countries [27], where the mutation frequency was estimated to be 50-71%. In Israel, however, a lower mutation frequency of approximately 27.5% was found [28]. In terms of the prevalence of MDR1 nt230(del4) among prone herding breeds, it is apparent that Rough Collies have the highest mutation rate across multiple countries.

For Australian Shepherds, approximately two-thirds of the evaluated dogs were obtained from two breeding kennels, with some individuals originating from private ownership. In this population, approximately 10 dogs had descended from the same parental pair. The mutant MDR1 allele frequency is similar to that found in the USA (16.60%) by Neff *et al*. [15]. This is not surprising because nearly all Australian Shepherds in our investigation had been imported or had parentage originating from the USA, except one dog that had been imported from Portugal. In addition, the mutation frequency noted here is consistent with that from a previous report from Brazil, where the frequency was 15.60% [18]. However, a higher mutation frequency and wide variation have been reported in various other countries, which may be attributed to the different sample sizes of those studies. As stated in large-scale MDR1 genotyping surveys, the mutation frequency was approximately 19.50-22% in Germany [21,22] and 29% in the USA [16], and a mutation frequency of approximately 25-46% has been reported in the small-to-medium-scale populations investigated in Israel [28], Japan [17], South Africa [19], Australia [20], and several European countries [24,26,27]. In terms of the universal distribution of MDR1 nt230(del4), the second most affected breed is most likely the Australian Shepherd. This has been noted in almost all previous investigations, and a similar trend was identified in Thailand. For Shetland Sheepdogs, this breed is the most popular herding dog breed in Thailand. About one-third of this population has originated from one breeding kennel that is free of MDR1 nt230(del4), whereas the origin of the remaining individuals was split equally between the other breeding kennel and private owners. Almost all dogs originated from the USA, with a small proportion originating from Canada and Russia. Our study suggests a slightly higher mutation frequency than that in the USA (7-8.4%) [15,16], as well as that reported in Japan [17] and Brazil [18] (1.2% and 7.9%, respectively). Much higher mutation frequencies, ranging from 23% to 39.3%, have been described in Israel [28] and numerous countries in Europe [21,22,24,26,27], other than Austria, where the frequency was 14.5% [27]. Based on the geographic distribution of MDR1 mutation, there is likely a high prevalence of homologous mutations in Shetland Sheepdogs across European countries.

For Old English Sheepdogs, the MDR1 genotypes were evaluated in very few samples because this breed is rarely found in Thailand. All samples were obtained from private owners, and they most likely originated from the USA with unrelated parentage. The mutation frequency noted in the current survey is slightly higher than those from the medium-to-large-scale studies in the USA, where the mutation frequency was 1.25-3.6% [15,16]. This discrepancy may be due to the small sample size in our study. In European countries, mutation frequencies reported in Germany [21,22] and Italy [26] varied from 4% to 7.5%, whereas a higher mutation frequency of approximately 11.5% has been described in UK [24]. In Brazil, however, the presence of MDR1 nt230(del4) was not identified in this breed [18]. These surveys provide clear evidence that the MDR1 nt230(del4) occurs at a comparably low prevalence in Old English Sheepdogs across multiple countries. In Border Collies, approximately two-thirds of the examined dogs originated from one breeding kennel, with some dogs sharing the same parental line, whereas the remaining individuals were privately imported from abroad, with unrelated parentage. The majority of these dogs originated from the USA, and a minority originated from Australia, France, Hungary, and the UK. Interestingly, we did not detect MDR1 nt230(del4) in any samples. This finding is similar to those of previous studies in Australia [20], Brazil [18], Belgium [25], and several European countries [27]. As stated in the large-scale study in the USA, a low mutation frequency of 1% was reported [16]. Therefore, it is most likely that the population of Border Collies in our survey originated from countries with a very low prevalence of the mutation, which had a significant impact on our results. Other than these surveys, a low mutation rate has also been observed in some European countries, including Germany [21,22], Italy [26], and the UK [24], with mutation frequencies of 1-3.5%. In addition, the presence of this mutation has been reported in South Africa [19] and Israel [28], with frequencies of 2.13% and 4.8%, respectively. On the basis of a herding breed genealogy study, the ancestor of Border Collies is speculated to have separated from the rest of ancestral population of old working collies in the UK and Ireland before the emergence of MDR1 nt230(del4) [15]. This may explain the remarkably low prevalence of MDR1 nt230(del4) in Border Collies compared to Rough Collies and other collie-related breeds.

The privately-owned German Shepherds assessed in this study were predominantly derived from the German line with different parentages, and only a very small proportion of dogs had descended from the American line. Because German Shepherds have been used extensively as working dogs, their breeding programs have been tightly controlled to improve specific desirable traits and eliminate some genetic defects over several decades. As expected, our investigation did not find any MDR1 nt230(del4) in any tested dogs. This result is similar to those from previous investigations in the UK [24], Belgium [25], and Italy [26]. However, the existence of the MDR1 nt230(del4) has been observed in some countries, including the USA [16], Israel [28], South Africa [19], and Brazil [29], with low mutation frequencies of 0.6-7.14%. In addition, we also investigated the MDR1 nt230(del4) in White Swiss Shepherds. These privately owned dogs had descended from two different parentages originating in Germany. In the present study, the mutant MDR1 allele was not detected in any samples. This finding is inconsistent with a previous report from Germany, which noted the MDR1 nt230(del4) occurred at a frequency of 13.5% [22]. Moreover, a wide range of mutation frequencies, from 7% to 24.2% [26,27], has been reported in several other European countries, with the lowest frequency being found in Italy [26] and the highest in Poland [27]. Interestingly, the occurrence of this mutation in White Swiss Shepherds has also been published in countries located outside the European zone, including South Africa [19] and Israel [28], where the mutation frequencies were 7.69% and 16.7%, respectively. As mentioned above, the population of White Swiss Shepherds in this investigation was very small, because this breed is not popular in Thailand. Therefore, it is important to note that our results may not reflect the true prevalence rate of the mutation, and the existence of this mutation in this breed cannot be ruled out. In addition, to study predisposed herding dog breeds, we investigated the MDR1 nt230(del4) in Thai Ridgebacks. This breed is known as a loyal family dog and originates from Thailand. It has been registered with the Fédération Cynologique Internationale (FCI) since 2004, and it is classified as a hunting dog (FCI standard number 338). To the best of our knowledge, no previous research into the prevalence rate of MDR1 nt230(del4) in this breed has been published as a reference. In the current study, about two-thirds of the examined dogs were obtained equally from two breeding kennels, with the rest of the individuals originating from private ownership, and most dogs were likely derived from unrelated parentage. Because the Thai Ridgebacks have not descended from the collie lineage and there is no documented evidence of toxicity related to P-glycoprotein substrates in this breed, it is generally assumed that this native breed is not predisposed to MDR1 nt230(del4). As expected, our results showed that there were no mutated MDR1 alleles in any Thai Ridgeback samples. Therefore, this finding provides strong evidence that the MDR1 nt230(del4) does not exist in Thai Ridgebacks. Furthermore, it is unlikely that a disease allele from the native breed will be passed to any mutation-prone breed through crossbreeding.

## Conclusion

This study describes the prevalence of MDR1 nt230(del4) in herding dog breeds in Thailand. The overall mutation rate of each breed is compatible with that from global reports. Nevertheless, due to the limited sample size, caution must be taken when interpreting these results. In addition, we explored for the first time the status of MDR1 genotypes in Thai Ridgebacks originating in Thailand. However, some limitations of this research should be acknowledged. First, our sample size was relatively small; thus, the estimated mutation rates are likely to be either under- or over-estimated. Second, we were unable to identify the ancestral origin of individual cases, and unintended selection bias may have occurred because the pedigree data for the evaluated dogs were only partially available. Therefore, further investigations should be conducted on a larger population, and a complete pedigree analysis should be performed to increase the impact of the research. Well-designed breeding programs should verify the MDR1 genotype in predisposed breeds. To minimize the presence of MDR1 nt230(del4) in the gene pool, parents used in breeding strategies should be free of the mutated MDR1 allele or should harbor at least one copy of the wild-type allele, whereas breeding between two individuals exhibiting the homozygous mutation should be avoided. Importantly, determination of the MDR1 status in suspected breeds is recommended before administering the P-glycoprotein substrates to prevent adverse drug reactions; this would also provide a useful guideline for the veterinarian in terms of adjusting the dosing regimen for individuals carrying the mutated allele.

## Authors’ Contributions

CL: Clinical examination of the animals, blood samples collection, study design, data interpretation, manuscript preparation, and revision. NP: Laboratory technical assistance and data interpretation. All authors have read and approved the final manuscript.
